# Assessing knowledge levels on coronavirus disease (COVID-19) among community members: The influence of community engagement efforts in Seke district, Zimbabwe: A cross-sectional study

**DOI:** 10.1371/journal.pone.0342318

**Published:** 2026-02-06

**Authors:** Enica Rutendo Chikanya, Moses John Chimbari

**Affiliations:** School of Nursing and Public Health, College of Health Sciences, Howard College Campus, University of KwaZulu-Natal, Durban, South Africa; University of Luzon, PHILIPPINES

## Abstract

**Introduction:**

The COVID-19 pandemic posed significant public health challenges globally, with effective containment relying heavily on community knowledge and engagement. This study assessed COVID-19 knowledge levels among community members in Seke District, Zimbabwe, and evaluated the influence of community engagement efforts.

**Methods:**

A community-based cross-sectional survey of 748 adults was conducted from January to March 2024 across rural, peri-urban, and farming settlements in Seke District, using structured interviews. Participants were selected using multistage cluster sampling and simple random sampling within clusters. Data were collected through structured face-to-face interviews using a pre-tested questionnaire assessing knowledge of COVID-19 (symptoms, transmission, prevention) and community engagement. Knowledge was scored on a 17-item questionnaire with a total possible score of 61 points; participants scoring 50% or more of the total possible score were classified as knowledgeable, a threshold based on prior similar studies. Data analysis included ANOVA, Student’s t-test, and post hoc tests with significance set at p < 0.05.

**Results:**

Only 27% scored above the knowledge threshold. Knowledge varied significantly by gender, age, residence, religion, education, and engagement with Village COVID-19 Taskforce Committees (p < 0.05). Males, younger adults, peri-urban residents, and those engaged with taskforce services showed higher knowledge levels.

**Conclusion:**

This study highlights notable knowledge gaps on COVID-19 in Seke District despite ongoing community engagement efforts. Unlike previous studies in the district, it uniquely links community engagement activities with knowledge outcomes across diverse demographic groups. Findings suggest the need for tailored, targeted public health education strategies to enhance health literacy and community participation. Strengthening inclusive community engagement can optimize pandemic preparedness and response in this and similar settings.

## Introduction

Community engagement (CE) involves the active participation of individuals, groups, and structures within defined social boundaries to inform decision-making, planning, governance, and service delivery [1,2]. The World Health Organization (WHO) defines CE as “a process of developing relationships that enable stakeholders to work together to address health-related issues and promote well-being to achieve positive health outcomes” [3].

The COVID-19 pandemic presented significant public health challenges globally, with particular impact in resource-limited settings such as Zimbabwe. Effective containment relied heavily on community knowledge, attitudes, and practices concerning the virus, influencing adherence to preventive measures such as hand hygiene, mask-wearing, and social distancing [4–7]. The COVID-19 pandemic underscored the critical role of CE in disseminating timely information and encouraging preventive behaviours [1,2]. The pandemic also revealed the critical importance of knowledge assessment and community engagement for effectively managing widespread disease outbreaks [1,8,9]. Evaluating knowledge levels related to COVID-19 among various communities is essential to tailor risk communication and intervention strategies effectively [10–14].

The intersection of community engagement with COVID-19 has been a topic of increasing importance and interest in recent years. Various studies have focused on understanding how different communities, including marginalized groups, have experienced COVID-19 response interventions and dissemination of COVID-19 information within their communities [1,8,9]. For instance, research indicates that 65% of marginalized communities reported inadequate access to reliable COVID-19 information, while 48% experienced barriers to participating in local health initiatives [1,8,9]. These metrics highlight critical gaps and underscore the need for targeted engagement strategies.

Previous research has emphasized the importance of assessing knowledge, attitudes, and practices (KAP) regarding COVID-19 among diverse populations and the role of CE in mitigating pandemic impacts [4–6]. Some studies have focused on specific groups such as university students [5], while others have explored the role of social media in risk communication during health crises [7]. Evaluations of knowledge, attitudes, and practices among different populations regarding COVID-19, as well as the role of community engagement in mitigating the impact of the pandemic emphasized the need for improved community engagement activities and effective communication during disease outbreaks [4–6].

The reported emphasis on the importance of understanding the knowledge levels and perceptions of different communities to develop effective risk communication strategies [10], aligns with work which emphasized the importance of sustaining community engagement, collaboration, and participation over time to build resilience and community power during and beyond the pandemic [6,11,12]. Other studies highlight the importance of understanding community-specific factors that influence behavior and engagement during a pandemic, emphasizing the role of communication strategies and state interventions in reducing the risks associated with the pandemic [13,14]. Effective management of disease outbreaks heavily relies on the knowledge and behaviors of community members, who are often the first line of defense against the spread of the virus [4–7].

Overall, existing literature emphasizes the significance of knowledge assessment to understand the influence of community engagement efforts in effectively managing the COVID-19 pandemic [4–7,10–14].

The COVID-19 pandemic had major public health challenges worldwide, particularly in 92 resource-limited settings such as Zimbabwe. As of April 13, 2024, over 260,000 cases and more than 5,700 deaths [15] were reported in Zimbabwe. Seke District alone recorded over 3,000 cases [16]. Effective outbreak management depends largely on community members’ knowledge, attitudes, and practices, which influence adherence to preventive measures [4–7]. Misconceptions or gaps in knowledge can hinder disease control efforts, increasing morbidity and mortality risks [4–7].

Prior investigations in Seke District, Zimbabwe, including those by Mutsaka-Makuvaza et al. [17] and Midzi et al. [18], have primarily assessed general awareness and knowledge of COVID-19 but gave limited attention to the influence of community engagement and its interaction with demographic variables. Understanding community-specific knowledge levels is essential for developing targeted information sharing strategies and promoting preventive behaviors.

This study uniquely evaluates how community engagement efforts influenced COVID-19 knowledge levels across diverse settlement types (rural, peri-urban, and farming) within Seke District. By capturing this nuanced understanding, the research extends existing knowledge and informs more targeted public health education and intervention strategies. Understanding these dynamics aligns with broader calls for sustained community engagement, collaboration, and participation to build resilience and community power during and beyond pandemics [6,11,12].

This approach allows for a nuanced understanding of how demographic factors and engagement activities interact to shape public health knowledge. Moreover, our detailed demographic and statistical analyses provide novel insights that extend existing knowledge. By addressing gaps identified in prior research, including limited consideration of community engagement’s role, this study contributes valuable evidence to inform targeted public health interventions.

Seke District in Zimbabwe, like many other regions, faced significant hurdles in controlling the disease, partly due to varying levels of awareness and understanding of COVID-19 among residents. Understanding what community members know about COVID-19 (its transmission, prevention, and treatment) is critical for designing effective public health interventions for future disease outbreaks [4–7]. Misconceptions or gaps in knowledge can hinder efforts to curb spread of diseases, risking increased morbidity and mortality, resulting in sub optimal health outcomes [4–7].

In Seke District, where a cumulative burden of over 3000 cases of COVID-19 was recorded [16], community engagement initiatives aimed to increase awareness and knowledge about COVID-19. However, the extent to which these efforts influenced knowledge levels remains underexplored. Understanding the relationship between community engagement and COVID-19 knowledge can inform public health strategies, optimize resource allocation, and enhance community-led interventions for current and future outbreaks.

This study aimed to assess COVID-19 knowledge levels among community members in Seke District, evaluate the extent and nature of community engagement efforts, and identify socio-demographic factors influencing knowledge. We hypothesized that: Community members actively engaged in COVID-19-related activities would demonstrate higher knowledge levels compared to those with little or no engagement,; Socio-demographic factors such as age, education level, and place of residence would significantly influence COVID-19 knowledge levels.

## Materials and methods

### Study design and setting

A community-based, cross-sectional study was conducted in three wards of Seke District to assess knowledge levels on COVID-19 and community engagement among community members (Wards 5, 11 and 13). The study was conducted from January to March 2024. Seke District is situated in the North-eastern part of Zimbabwe, approximately 52 km South of Harare, the capital city of Zimbabwe. Seke District is easily accessible from metropolitan Harare thus making it susceptible to cross border transmission of communicable diseases like COVID-19. The district has eight government health facilities, four council clinics and three private clinics that provide healthcare services to a catchment area population of over 200,000 [19]. In addition, the study design employed rigorous multistage sampling and validated knowledge assessment tools, enhancing reliability and representativeness compared to some earlier investigations.

### Sampling

**Fig 1:**
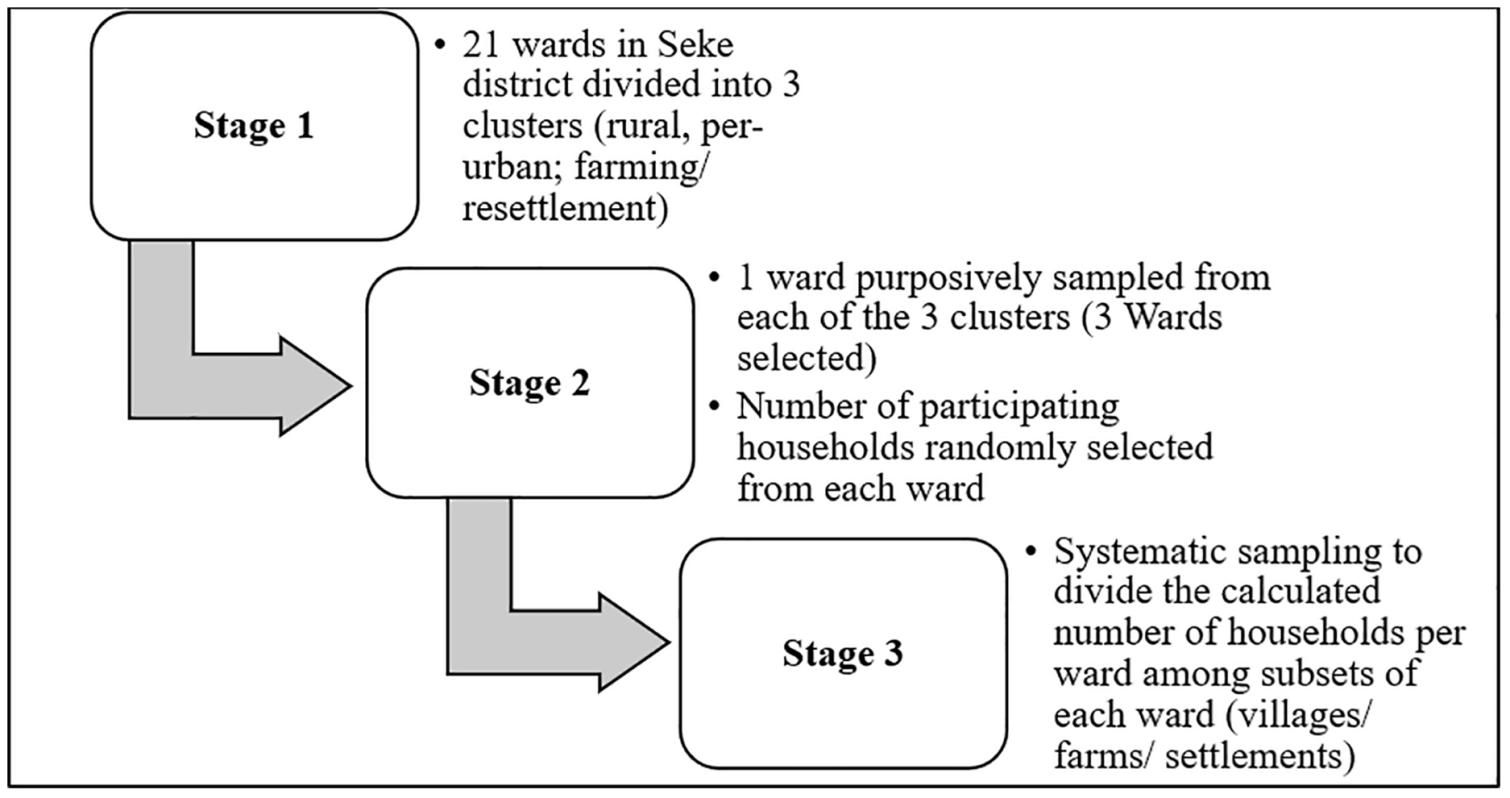
Multistage sampling for selecting study sites for household level knowledge assessment.

Seke District was selected due to its diverse settlement types, close proximity to Harare and high COVID-19 burden, making it representative for assessing community engagement impact. Multi-stage sampling was employed. Wards were grouped into three clusters. One ward was purposively selected from each cluster based on highest COVID-19 case burden. Cluster sampling ensured selection of every geographical cluster in Seke District. Each cluster had an equal chance of being selected. This resulted in representation of diverse settlements. The first sampling stage involved dividing the twenty-one (21) wards of Seke District into three clusters namely rural, peri urban and farming/ resettlement to make sure that different populations are included in the study. One (1) ward was purposively selected from each of the 3 clusters to come up with three wards from which a sample size of interviewed household members was calculated using simple random sampling. A total of seven hundred and forty-eight households were selected from the three wards based on a 95% confidence interval and 5% margin of error. Fig 1 is a graphical representation of the different stages of sampling.

### Participant selection

The source population was all the residents of Seke District, and the study population was all selected adults in the selected wards (lowest administrative unit) in the rural, peri-urban and resettlement/farming wards. The following inclusion criteria were applied in this study: adults (i.e., older than 18 years of age) of any sex, formal residents of Seke District, and willing to participate indicated by signing of an informed consent. 748 individuals were approached and examined for eligibility. They all agreed to be screened for eligibility. 748 individuals met the inclusion criteria (residing in Seke District and aged 18 and above). 748 eligible individuals consented and were enrolled. The sample size estimation was calculated using a single population formula with a 5% margin of error and 95% confidence level. Random sampling was used to select the study participants. One ward from each settlement type was selected using purposive sampling, based on COVID-19 case burden, where the wards with the highest number of cases were selected from each cluster. Finally, the sampling with population proportional to the size was calculated for the study participants for each selected ward to attain the total sample size by using the proportionate allocation formula. The sampling details are in Table 1.

**Table 1:** Sample size by ward.

Cluster	Ward number	Total households	Study sample size
Rural	**5**	892	269
Farming/ Resettlement	**11**	321	176
Peri urban	**13**	1420	303
**Total**		**2633**	**748**

Sample proportion for each ward is the ratio of the number of household representatives aged eighteen (18) years and above in the ward to the total number of households in the three selected wards.

### Data collection

The data were obtained from the consenting participants through face-to-face interviews using a structured questionnaire on knowledge levels on COVID-19 and community engagement. The questionnaire was adopted from previous studies [20–22] and pre-tested. It had ten distinct sections, two of which focused on assessing knowledge levels on COVID-19 and knowledge levels on community engagement. The data were collected from January 2024 to February 2024.

### Outcome variables

The knowledge of the respondents was assessed using a 17-item questionnaire, developed using various literature sources [23–25]. The questions included the clinical characteristics of the disease (i.e., primary symptoms, availability and effectiveness of treatment, and severity), addressed transmission (i.e., infection through contact with animals and transmission through respiratory droplets), and prevention and control (i.e., wearing medical masks for prevention), definition of community engagement and purposes of community engagement. All respondents could respond with “Yes,” “No,” or “Don’t know.” For some questions, respondents chose the correct options in response to the questions. The knowledge scores were calculated by assigning one point to each correct answer, and an aggregate score was calculated (range 0–61). It was measured by calculating the total of all correct answers to the 17 questions. Participants were categorized as knowledgeable, if they scored at least 50% of the total possible score, or not knowledgeable, if they scored less than 50% of the possible score, consistent with thresholds used in comparable knowledge assessment studies [23–25].

### Data sources/ management

Table 2 shows variables of interest, data sources, and methods of assessment.

**Table 2:** Variables of interest, data sources, and methods of assessment.

Variable	Source of data	Methods of assessment/measurement	Comparability across groups	Efforts to address bias
**Knowledge Level of COVID-19**	Community-based surveys, questionnaires	Structured questionnaires with multiple-choice and Likert-scale questions assessing knowledge of COVID-19 transmission, symptoms, prevention, treatment, and community engagement concepts	Standardized questionnaire administered to all groups	Pilot testing for clarity; training of data collectors; random sampling within communities to minimize selection bias
**Community Engagement Efforts**	Community-based surveys, questionnaires	Structured questionnaire with multiple-choice questions assessing receipt of community engagement services from village COVID-19 taskforce committee members	Standardized questionnaire administered to all groups	Use of uniform definitions and consistent interview protocols

### Addressing potential sources of bias

To address selection bias, random sampling techniques were employed within each selected ward, as shown in table 1.

Information bias was prevented by using a validated, adapted questionnaire [26,27]. Training of data collectors was thoroughly done before commencement of data collection [28]. Anonymity was ensured to reduce social desirability bias [29]. To address recall bias, the recall period in questions was limited [30,31]. Participants were prompted with specific questions to improve accuracy. Interviewer bias was addressed by standardizing data collector training, use of structured interviews and data collection protocols [32,33].

### Data quality control

On the survey instrument, omit or skip patterns were utilized to properly direct respondents to relevant questions. Data verification processes, included the spot-checking of the questionnaire at the data-gathering sites, which were implemented to ensure the accuracy of the data.

### Data analysis

The data were entered in KOBO Collect, and analyzed by Microsoft Excel. A Kolmogorov-Smirnov test was used to check for the normal distribution of data. Student T test was used to analyse variance of knowledge levels between males and females. Analysis of variance (ANOVA) (one-way) was used to compare knowledge levels and check for differences between and within groups. Knowledge levels of the participants were compared by various demographic factors (gender, age, place of residence, religion, level of education, and receipt of services from village COVID-19 taskforce committees). Univariate analysis was used for analysis of socio-demographic data and knowledge levels on the subject of COVID-19 and community engagement. Post hoc tests (Cohen’s d and Tukey HSD) were carried out to identify exactly which groups differed from each other. The data were accessed for research purposes on 1 March 2024. Authors had no access to information that could identify individual participants during or after data collection.

Descriptive statistics were computed to summarize the demographic characteristics of the study population, including means and standard deviations for continuous variables and frequencies and percentages for categorical variables.

Inferential analysis was done to compare knowledge levels across different groups, one-way Analysis of Variance (ANOVA) was employed. Post-hoc analyses with Tukey’s HSD were conducted to identify specific group differences where ANOVA indicated significance.

Potential confounders such as age, gender and education level, were identified based on prior literature and bivariate analyses. Subgroup analyses were conducted to evaluate differences in COVID-19 knowledge among key demographic categories (age groups, gender, education levels).

Missing data were assessed for patterns and mechanisms. The proportion of missing data was less than 5% and missing completely at random (MCAR), therefore, listwise deletion was employed. This method was used because the data were missing at random, listwise deletion was simple, and unbiased estimates were provided, consistent with best practices for handling low MCAR missingness. In terms of sampling strategy and analytical approach, all analyses were conducted using survey procedures in statistical software, which ensured unbiased estimates and correct standard errors that reflect the sampling design. Sensitivity analysis was undertaken to assess the robustness of findings, by repeating analyses.

### Ethical considerations

The study obtained ethics approval from the Humanities and Social Sciences Research Ethics Committee (HSSREC) of the University of KwaZulu Natal (HSSREC/00005706/2023) and the Medical Research Council of Zimbabwe (MRCZ/A3107). Written permission to conduct the study was obtained from the Provincial Medical Director of Mashonaland East Province, Zimbabwe. Letters of approval were written by the two ethics committees. Participation was voluntary and each participant gave written informed consent after a clear explanation of the research objectives, before taking part. The participants were assured that all responses would remain confidential. The right to withdraw from the study was respected. The recruitment period for this study was 12 January 2024 to 21 January 2024.

### Patient and public involvement

We involved the public in at the survey development stage, where members of the community took part in a pilot survey after development of the questionnaire. Their feedback helped refine the language and appropriateness of the questions, enhancing clarity for diverse literacy levels. Public involvement significantly enhanced the research’s relevance, accuracy, and community buy-in. The insights gained during interviews revealed critical misconceptions about COVID-19 that were previously unrecognized by researchers. This collaborative approach increased the credibility of our findings and suggested tailored interventions that resonate with community needs.

Furthermore, engaging community members in the research process fostered trust between researchers and participants, making them more likely to support and engage with subsequent public health initiatives. We are committed to sharing our findings with the community through accessible formats. We will present the results in community meetings. We will also collaborate with relevant stakeholders to integrate findings into health campaigns aimed at improving knowledge and engagement regarding COVID-19 and other public health issues.

## Results

A total of 2633 eligible community members were identified through household surveys and secondary data from the population census which was carried out in 2022 [19]. 747 participants completed the questionnaire interviews by answering all questions. Data from 747 participants were included in the final analysis. Data from 1 participant was not complete. There were no refusals to participate in the study. Fig 1 is a flow diagram depicting participant selection progression throughout the study stages (Fig 1: Flow diagram depicting participant selection).

The study achieved a high response rate of 99.9%, enrolling 748 participants from diverse settings in Seke District, including rural (35.9%), resettlement/farming (23.5%), and peri-urban areas (40.5%). Overall, knowledge about COVID-19 was limited, with 73% scoring below 50% on a 17-item questionnaire assessing symptoms, transmission, prevention, and community engagement. Knowledge levels varied significantly across demographic groups. Receipt of services from Village COVID-19 Taskforce Committees (VCCs) was also associated with significantly higher knowledge levels. These findings highlight disparities in COVID-19 understanding linked to demographic and community engagement factors.

### Socio-demographic characteristics of participants

The sociodemographic characteristics of the study participants are presented in Table 3.

**Table 3:** Socio-demographic characteristics of study participants, Seke District residents, North-eastern Zimbabwe.

Variable	Category	Frequency	Percentage (%)
**Gender**	Female	486	65
	Male	262	35
	**Total**	748	100
**Marital status**	Married/Cohabiting	596	79.7
	Widowed	55	7.4
	Never married/Single	53	7.1
	Divorced/Separated	41	5.5
	**Total**	745	99.7
**Highest level of education**	Secondary	539	72.1
	Technical/Vocational	108	14.4
	Primary	36	4.8
	Graduate	31	4.1
	No formal education	29	3.9
	Masters and above	1	0.1
	**Total**	744	99.4
**Religion**	Christianity	727	97.2
	Other	15	2
	Islam	2	0.3
	**Total**	744	99.5
**Main source of income**	Crop farming	250	33.4
	Self employed	204	27.3
	Labour/ Employment	176	23.5
	Other	47	6.3
	Petty trade	41	5.5
	Remittance	22	2.9
	Livestock	3	0.4
	**Total**	745	99.3

Out of the 748 community residents that the study reached, 748 participants consented and 747 responded to all questions in the questionnaire, with a response rate of 99.9%. One participant opted not to respond to all of the questions. Regarding place of residence, 269 (35.9%) were from rural areas, 176 (23.5%) were from resettlement/ farming area and 303 (40.5%) were from the peri-urban area. The mean age of the participants was 41.29 ± 13.66 SD. The majority of the participants were female [486 (65%)] and had substantial sources of income [630 (84.2%)]. Regarding their educational status, the majority reached secondary school level (539) (72.1%). Regarding potential confounders, gender may influence receipt of community engagement services and knowledge levels; level of education impacts understanding of COVID-19 information; source of income, which reflects socioeconomic status affects access to information. Socioeconomic factors and education level, are potential confounders in assessing the relationship between community engagement and COVID-19 knowledge. Three participants (0.4%) had missing information on marital status; four participants (0.5%) had missing information on level of education; 0.5% of the participants had missing information for religion; five participants (0.7%) of the participants had missing information on source of income.

### Knowledge levels

Seventeen questions were used to measure the knowledge of the study participants regarding COVID-19 and community engagement. The mean score from the responses of participants was 44(SD = 8.72, range 0–72). 73% of the participants scored less than 50% of the possible mark. Notably, the participants had some knowledge about COVID-19. However, in the case of the most commonly known facts about COVID-19, the aggregate scores were low for 73% of the respondents since they did not obtain full marks in all of the questions. Most of the participants (96%) knew the definition of COVID-19. The most common symptoms of COVID-19 which were known to the participants were dry cough (67%) and fever (62%). The other less common COVID-19 symptoms which were known by the study participants were headache (78%), sore throat (66%) and loss of taste of smell (47%), among many others which were reported by fewer participants. Respiratory failure was the most common complication of COVID-19 that participants were aware of (71%). Very few respondents knew about thromboembolism and/or multiorgan failure, including injury of the heart, liver or kidneys (19%) acute respiratory distress syndrome (ARDS) (18%) and sepsis and septic shock (4%). The population at risk of contracting COVID-19 was reported as those with underlying medical problems like high blood pressure, heart and lung problems, diabetes, obesity or cancer by 72% of the participants. The other reported population groups at risk were people aged 60 years and over (65%). It was also reported that anyone could get sick with covid-19 and become seriously ill or die at any age (29%). The most common prevention measure for COVID-19 reported by the respondents was wearing a mask (87%). Many of the respondents were knowledgeable about when to get tested for COVID-19 (86%), what to do when exposed to a COVID-19 patient (61%) and vaccination as a COVID-19 prevention measure. 90% of the participants had general knowledge about community engagement related to COVID-19.

### Comparison of knowledge levels across demographic factors

Knowledge levels of the participants were compared by various demographic factors (gender, age, place of residence, religion, level of education, and receipt of services from village COVID-19 taskforce committees). Table 4 shows results for analysis of variance and Table 5 shows results for post hoc tests (Tukey HSD).

**Table 4:** Analysis of variance (ANOVA) of COVID-19 knowledge scores by demographic factors.

Factor	Groups	N	Mean Knowledge Score	SD	F	p-value
**Gender** ^**1**^	Male	262	40	7.88	–	0.00075^1^
	Female	486	38	8.45		
**Age Group** ^**2**^	18–24 (Young adults)	96	41.28	7.95	11.42	<0.000001²
	25–39 (Adults)	289	39.90	7.63		
	40–59 (Middle-aged)	262	38.99	8.41		
	60–74 (Older adults)	90	34.64	7.24		
	75+ (Seniors)	6	30.46	12.15		
**Place of Residence** ^**2**^	Ward 5 (Rural)	269	36.49	4.63	292.82	<0.000001²
	Ward 11 (Farming)	176	49.65	7.29		
	Ward 13 (Peri-urban)	303	47.73	7.45		
**Religion** ^**2**^	Christianity	727	39.19	8.19	8.05	0.00035²
	Islam	2	43.10	2.44		
	Other	19	31.64	9.84		
**Education Level** ^**2**^	No formal education	29	30.80	6.32	20.69	<0.000001²
	Primary	36	42.24	8.31		
	Secondary	539	38.04	8.10		
	Technical/Vocational	108	43.59	6.45		
	Graduate	31	44.64	4.60		
**Receipt of VCC Services** ^**2**^	Yes	517	42.13	7.02	176.09	<0.000001²
	No	218	32.14	5.56		
	Don’t know	8	35.06	6.98		

¹Student’s t-test for gender.

²One-way ANOVA for other factors.

**Table 5:** Summary of Tukey HSD post-hoc test results for significant group comparisons.

Factor	Group Comparison	p-value	Significance
**Age Group**	18–24 vs 60–74	0.0010	** (p < 0.01)
	18–24 vs 75+	0.0115	* (p < 0.05)
	25–39 vs 60–74	0.0010	** (p < 0.01)
	25–39 vs 75+	0.0356	* (p < 0.05)
**Place of Residence**	Ward 5 vs Ward 11	0.0010	** (p < 0.01)
	Ward 5 vs Ward 13	0.0010	** (p < 0.01)
	Ward 11 vs Ward 13	0.0065	** (p < 0.01)
**Religion**	Christianity vs Other	0.0010	** (p < 0.01)
**Education Level**	Graduate vs No Formal Education	0.0010	** (p < 0.01)
	Graduate vs Secondary	0.0010	** (p < 0.01)
	No Formal Education vs Primary	0.0010	** (p < 0.01)
	No Formal Education vs Secondary	0.0010	** (p < 0.01)
	No Formal Education vs Technical	0.0010	** (p < 0.01)
	Primary vs Secondary	0.0141	* (p < 0.05)
	Secondary vs Technical/Vocational	0.0010	** (p < 0.01
**Receipt of VCC Services**	Received vs No	0.0010	** (p < 0.01)
	Received vs Don’t know	0.0087	** (p < 0.01)

### Gender

To compare knowledge levels, a Student T test was carried out. The recorded p-value (0.000751296) was much lower than 0.05, therefore we rejected the null hypothesis. The Effect Size (Cohen’s d) was measured to determine how meaningful the difference was and to better understand the magnitude of the difference in knowledge levels between the genders. The Cohen’s ds result was 0.254658898. Males had a higher mean knowledge level (40) compared to females (38). This suggests that, on average, males demonstrate a higher level of knowledge regarding community engagement in response to COVID-19. The standard deviation for females (8.45) was slightly higher than that for males (7.88), indicating that the knowledge levels among females may be more spread out or varied compared to males. Overall, the results suggest that there is a significant difference in knowledge levels regarding community engagement in response to COVID-19, with males showing higher knowledge levels than females.

### Age group

There were significant differences among the five age groups. The F-value (11.42173) was significantly greater than the F critical value (2.383999), and the p-value (5.33E-09) indicates that there are statistically significant differences in the knowledge levels about community engagement regarding COVID-19 across the five age groups. Tukey HSD results in Table 5

strongly suggested that one or more pairs of groups are significantly different. The 18–24 years (young adults) – 75 and older (seniors) and the 25–39 years (adults) −75 and older (seniors) age group pairs had statistically significant differences, with p values which are less than 0.05. The 18–24 years (young adults) – 60–74 (older adults) and the 25–39 years (adults) – 60–74 (older adults) pairs also had statistically significant differences with p values which were less than 0.01.

### Place of residence

The extremely low p-value of 1.47E-94 indicates a significant difference in knowledge levels among the groups. Results for a further post-hoc test (Tukey HSD) in Table 5, indicated p values less than the alpha level 0.05, hence the differences between the three stated pairs (Ward 5 vs Ward 11; Ward 5 vs Ward 13; Ward 11 vs Ward 13) were statistically significant.

### Religion

The one-way ANOVA results indicated that there were statistically significant differences in knowledge levels among the three religious groups. Post-hoc test (Tukey’s HSD) tests revealed that there was a statistically significant difference between Christians and community members of other religions (Table 5). Insignificant statistical differences were seen between Christians and Islams and between Islams and community members of other religions.

### Level of education

The F-statistic of 20.6869, along with a very low p-value (2.27E-19), indicated that there was a significant difference in knowledge levels across the different educational levels. Post hoc test (Tukey’s HSD) results in Table 5 showed statistically significant differences among 7 pairs of levels of education (Graduate vs No formal education; Graduate vs Secondary; No formal education vs Primary; No formal education vs Secondary; No formal education vs Technical/ Vocational; Primary vs Secondary; Secondary vs Technical/ Vocational). The other 3 pairs had insignificant differences.

### Receipt of VCC services

The low p-value (2.79E-63) indicates a significant difference in knowledge levels among the three groups of community members. Post hoc Tukey HSD results in Table 5 showed that there were statistically significant differences between community members who did not know whether they received VCC services or not, and those who received the services, as well as between those who did not receive VCC services and those who received the services. There was no statistically significant difference between those who did not know whether they received VCC services and those who did not receive VCC services.

## Discussion

The study provides important insights into COVID-19 knowledge levels among community members in Seke District, Zimbabwe, revealing significant gaps despite ongoing community engagement efforts. Only 27% of participants demonstrated adequate knowledge, underscoring persistent challenges in health literacy within this population. These findings are consistent with prior research in Zimbabwe and the broader Southern African region, where studies have reported variable but often limited KAP related to COVID-19, particularly in rural and peri-urban communities [17,18,34,36]. The study found that demographic characteristics and community engagement efforts influenced knowledge retention and understanding of COVID-19. The findings underscore the importance of tailored educational strategies that consider demographic disparities and engagement levels, thereby offering actionable recommendations for public health policy in Zimbabwe and similar settings

In Zimbabwe, several surveys conducted during the pandemic have highlighted inadequate understanding of COVID-19 transmission and prevention measures, often compounded by misinformation and resource constraints [17,18]. Our study confirms these trends but extends them by explicitly linking knowledge disparities to demographic factors such as gender, age, education level, place of residence, and importantly, engagement with Village COVID-19 Taskforce Committees (VCCs). This connection between community engagement efforts and knowledge outcomes has been underexplored in prior Zimbabwean research, making a key contribution of this study.

Regionally, similar KAP assessments in neighboring countries like Ethiopia, South Africa, and Zambia have documented the critical role of community-based interventions and trusted local structures in enhancing awareness and compliance [8,34,37]. Our findings resonate with this broader evidence base, emphasizing that active participation in community health initiatives correlates with higher COVID-19 knowledge scores. Notably, participants who reported receiving services from VCCs exhibited significantly greater knowledge, supporting the value of grassroots engagement models in pandemic response.

The observed demographic disparities align with regional patterns where younger adults, males, and those with higher education tend to have better COVID-19 knowledge [6,34]. This highlights the need for tailored messaging and outreach that addresses the specific barriers facing women, older adults, and individuals with lower education levels in Zimbabwe. For example, digital information campaigns may effectively reach younger populations, while community meetings and printed materials could better serve other groups.

This study’s unique contribution lies in its comprehensive assessment across diverse settlement types (rural, peri-urban, and farming/resettlement areas) within a single district, combined with rigorous multistage sampling and validated knowledge instruments. Such granularity allows for nuanced understanding of how demographic and engagement factors intersect to shape knowledge outcomes, informing more targeted public health strategies.

The study had several strengths. Having a focus on community engagement, the study highlighted the role of community involvement in knowledge dissemination, which is crucial for pandemic response strategies. The local context of the study is another strength. Conducting the study within the specific socio-cultural setting of Seke District allowed for tailored insights relevant to the community’s unique challenges and responses. Furthermore, the representation of all three clusters of Seke district allowed for contextual reflections from participants residing in different settings (rural, peri-urban and farming areas).

However, temporal limitations were noted. As the study was conducted during a specific period after the pandemic, findings may not reflect changes in knowledge levels as the situation evolved or as new information emerged. Reliance on self-reported measures can introduce bias, as participants may have overstated their knowledge levels due to social desirability.

The findings from our study indicate that a substantial proportion of the interviewed community members in Seke district exhibited low levels of knowledge regarding COVID-19, with notable disparities linked to socio-demographic factors as evidenced by analysis of variance (ANOVA) analyses. This suggests that targeted community engagement efforts may need to be intensified or tailored to address specific demographic groups that are less informed. However, these results should be interpreted with caution due to some considerations. The cross-sectional design limits causal inferences; the observed outcomes do not necessarily imply that community engagement efforts directly influenced knowledge levels, and other unmeasured factors could be contributing. While the study underscores important gaps in knowledge and disparities linked to demographic factors, cautious interpretation is essential, and further studies are warranted to validate these findings and inform tailored intervention strategies.

In comparison to similar studies conducted in different regions, this research has shown differences in knowledge levels based on community engagement interventions [34–39]. The evidence from the previous studies suggests that community health outreach services play a critical role in enhancing health knowledge across different populations [34–39]. Participants who engaged with these services demonstrated improved health understanding. Ongoing access to these resources is essential for sustaining knowledge and promoting better health outcomes. The difference in community engagement strategies might explain disparities in knowledge levels [34–39]. While some studies identified misinformation as a challenge, the Seke District study emphasized active community engagement as a mitigation factor, showcasing an effective grassroots approach that was less prominent in other studies.

The identification of knowledge gaps presents an opportunity for responsible authorities to tailor educational campaigns aimed at increasing public knowledge about COVID-19 and other public health events which may occur in the future, as well as the component of community engagement. For instance, younger individuals may benefit from digital education strategies that leverage social media platforms, while older populations might require more traditional methods, such as community meetings or printed materials. Interventions can be further tailored to religious, social and cultural norms as well.

Furthermore, the discrepancy between basic knowledge and a comprehensive understanding leads to potential risks in adhering to health guidelines and limited community participation. Studies by several authors indicated that incomplete knowledge can lead to increased anxiety and lower compliance with health measures [40–43]. Therefore, educational initiatives should focus on enhancing understanding of the science behind community engagement in relation to COVID-19 response, alongside promoting compliance with recommended preventive measures.

Several questions remain unanswered following this study. Future research could explore the long-term effects of community engagement on knowledge retention and behaviour change. Investigating how different socioeconomic factors influence knowledge levels across diverse communities would also be valuable. Additionally, further studies could assess community knowledge and perception, as well as evaluate the effectiveness of different community engagement interventions. Understanding these dynamics will be crucial for preparing for future public health initiatives and ensuring that communities are resilient in the face of new health challenges. Future research should also explore strategies for effectively bridging this knowledge divide, helping to cultivate a populace that is not only aware but genuinely informed about infectious diseases, and empowered to fully participant in public health emergency response interventions.

In terms of external validity, the sample included a diverse cross-section of community members in terms of age, gender, socioeconomic status, education, and place of residence, the findings are more likely to be applicable to the broader community in Seke District. However, if the sample was limited to specific subgroups or those easily accessible, the external validity may be constrained. The probability sampling technique employed (random sampling) enhances the representativeness of the sample, thereby increasing generalisability.

Cultural norms, beliefs, and socioeconomic conditions unique to Seke District may limit the generalisability to other districts within Zimbabwe or to similar settings elsewhere. Differences in community engagement strategies or health communication channels may further influence external validity. The use of standardized, validated questionnaires and reliable data collection procedures enhances the potential for the results to be applicable beyond the original study setting.

## Conclusion

This study assessed knowledge levels on COVID-19 among community members, in relation to community engagement efforts in the COVID-19 response in Seke District, Zimbabwe. The study identified statistically significant differences based on gender, age, place of residence, religion, educational attainment, and the receipt of services from village COVID-19 taskforce committees (VCCs). The contributions of this study highlight the need for targeted educational interventions that consider these demographic factors to enhance community engagement and improve public health emergency responses. The findings suggest that tailoring communication strategies and resource distribution based on these variables could lead to more effective COVID-19 response efforts and foster greater community resilience in future public health crises.

Overall, the results suggest that there is a significant difference in knowledge levels regarding community engagement in response to COVID-19. Further analyses to investigate the reasons behind the difference in knowledge levels; exploration of other demographic factors that might influence these results; qualitative assessment to understand the context of knowledge and further engagement, may be considered.

The statistically significant differences in knowledge levels across these demographic factors highlight the complexity of public health communication, particularly during a crisis like COVID-19. Effective strategies must be multi-faceted and consider the unique needs of different demographic groups. Tailoring engagement efforts to address these disparities can enhance community resilience and participation, ultimately contributing to a more effective public health response.

By understanding these implications, responsible authorities can implement more inclusive, responsive, and effective community engagement strategies that meet the diverse needs of the populations they serve.

### Implications for public health education

The identification of knowledge gaps presents an opportunity for responsible authorities to tailor educational campaigns aimed at increasing public knowledge about COVID-19 and other public health events which may occur in the future, as well as the component of community engagement. For instance, younger individuals may benefit from digital education strategies that leverage social media platforms, while older populations might require more traditional methods, such as community meetings or printed materials. Interventions can be further tailored to religious, social and cultural norms as well.

Furthermore, the discrepancy between basic knowledge and a comprehensive understanding leads to potential risks in adhering to health guidelines and limited community participation. Studies by several authors indicated that incomplete knowledge can lead to increased anxiety and lower compliance with health measures [40,42–44]. Therefore, educational initiatives should focus on enhancing understanding of the science behind community engagement in relation to COVID-19 response, alongside promoting compliance with recommended preventive measures.

### Recommendations

Addressing these knowledge gaps is essential for improving health literacy and informed participation in health-related decision-making. Future research should explore strategies for effectively bridging this knowledge divide, helping to cultivate a populace that is not only aware but genuinely informed about infectious diseases, and empowered to fully participant in public health event response interventions.

## Supporting information

S1 File Questionnaire on inclusivity in global research. (DOCX)

S2File Data 1. (XLSX)

S3 File Data 2. (XLSX)

S4 File Data 3. (XLSX)

S5 File STROBE checklist. (DOCX)

S6 File Fig 2. (TIF)
