# Supplementary material for: Assessing knowledge levels on coronavirus disease (COVID-19) among community members: The influence of community engagement efforts in Seke district, Zimbabwe: A cross-sectional study
Source: PLoS One. 2026 Feb 6;21(2):e0342318. doi: 10.1371/journal.pone.0342318 (PMC12880637; doi:10.1371/journal.pone.0342318)
Supplement: S5 File — (DOCX) [file pone.0342318.s005.docx]

STROBE Statement—Checklist of items that should be included in reports of ***cross-sectional studies***

|  | Item No | Recommendation |  |
| --- | --- | --- | --- |
| **Title and abstract** | 1 | (*a*) Indicate the study’s design with a commonly used term in the title or the abstract | Page 2, line 25 |
|  |  | (*b*) Provide in the abstract an informative and balanced summary of what was done and what was found | Page 2, lines 30 to 46 |
| Introduction | | |  |
| Background/rationale | 2 | Explain the scientific background and rationale for the investigation being reported | Page 5, lines 98 to 112 |
| Objectives | 3 | State specific objectives, including any prespecified hypotheses | Pages 5 to 6, lines 113 to 120 |
| Methods | | |  |
| Study design | 4 | Present key elements of study design early in the paper | Page 6, line 124 |
| Setting | 5 | Describe the setting, locations, and relevant dates, including periods of recruitment, exposure, follow-up, and data collection | Page 6, lines 126 to 132 |
| Participants | 6 | (*a*) Give the eligibility criteria, and the sources and methods of selection of participants | Page 7, lines 147 to 162 |
| Variables | 7 | Clearly define all outcomes, exposures, predictors, potential confounders, and effect modifiers. Give diagnostic criteria, if applicable | Pages 8 to 9, lines 173 to 184 |
| Data sources/ measurement | 8* | For each variable of interest, give sources of data and details of methods of assessment (measurement). Describe comparability of assessment methods if there is more than one group | *Page 9, lines 187 to 189* |
| Bias | 9 | Describe any efforts to address potential sources of bias | Pages 9 to 10, lines 191 to 198 |
| Study size | 10 | Explain how the study size was arrived at | Page 7, lines 158 to 159 |
| Quantitative variables | 11 | Explain how quantitative variables were handled in the analyses. If applicable, describe which groupings were chosen and why | Page 9, lines 194 to 203 |
| Statistical methods | 12 | (*a*) Describe all statistical methods, including those used to control for confounding | Page 10 lines 206 to 210; Page 11, lines 216 to 221 |
|  |  | (*b*) Describe any methods used to examine subgroups and interactions | Page 11, 219 to 221 |
|  |  | (*c*) Explain how missing data were addressed | Page11, lines 226 to 227 |
|  |  | (*d*) If applicable, describe analytical methods taking account of sampling strategy | Page 11, lines 227 to 230 |
|  |  | (*e*) Describe any sensitivity analyses | Page 11, lines 230 to 231 |
| Results | | |  |
| Participants | 13* | (a) Report numbers of individuals at each stage of study—eg numbers potentially eligible, examined for eligibility, confirmed eligible, included in the study, completing follow-up, and analysed | Page 13, lines 261 to 271 |
|  |  | (b) Give reasons for non-participation at each stage | Page 13, line 269 |
|  |  | (c) Consider use of a flow diagram | Page 13, 269 |
| Descriptive data | 14* | (a) Give characteristics of study participants (eg demographic, clinical, social) and information on exposures and potential confounders | Page 14, lines 274 to 281; Page 15, lines 286 to 290 |
|  |  | (b) Indicate number of participants with missing data for each variable of interest | Page 15, lines 290 to 294 |
| Outcome data | 15* | Report numbers of outcome events or summary measures | Page 15, lines 297 to 306; Page 16, lines 307 to 319 |
| Main results | 16 | (*a*) Give unadjusted estimates and, if applicable, confounder-adjusted estimates and their precision (eg, 95% confidence interval). Make clear which confounders were adjusted for and why they were included | Not applicable, Analysis of variance (ANOVA) was used for analysis |
|  |  | (*b*) Report category boundaries when continuous variables were categorized | Page 14, line 275 to 277 |
|  |  | (*c*) If relevant, consider translating estimates of relative risk into absolute risk for a meaningful time period | Not relevant |
| Other analyses | 17 | Report other analyses done—eg analyses of subgroups and interactions, and sensitivity analyses | Page 11, lines 230 to 231 |
| Discussion | | |  |
| Key results | 18 | Summarise key results with reference to study objectives | Page 23, lines 424 to 429 |
| Limitations | 19 | Discuss limitations of the study, taking into account sources of potential bias or imprecision. Discuss both direction and magnitude of any potential bias | Page 24, lines 436 to 440 |
| Interpretation | 20 | Give a cautious overall interpretation of results considering objectives, limitations, multiplicity of analyses, results from similar studies, and other relevant evidence | Page 24, lines 443 to 454 |
| Generalisability | 21 | Discuss the generalisability (external validity) of the study results | Page 26, lines 493 to 504 |
| Other information | | |  |
| Funding | 22 | Give the source of funding and the role of the funders for the present study and, if applicable, for the original study on which the present article is based | Not applicable, no funding was provided for the present study |

*Give information separately for exposed and unexposed groups.

**Note:** An Explanation and Elaboration article discusses each checklist item and gives methodological background and published examples of transparent reporting. The STROBE checklist is best used in conjunction with this article (freely available on the Web sites of PLoS Medicine at http://www.plosmedicine.org/, Annals of Internal Medicine at http://www.annals.org/, and Epidemiology at http://www.epidem.com/). Information on the STROBE Initiative is available at www.strobe-statement.org.
