# Supplementary figures and images for: Assessing knowledge levels on coronavirus disease (COVID-19) among community members: The influence of community engagement efforts in Seke district, Zimbabwe: A cross-sectional study
Source: PLoS One. 2026 Feb 6;21(2):e0342318. doi: 10.1371/journal.pone.0342318 (PMC12880637; doi:10.1371/journal.pone.0342318)

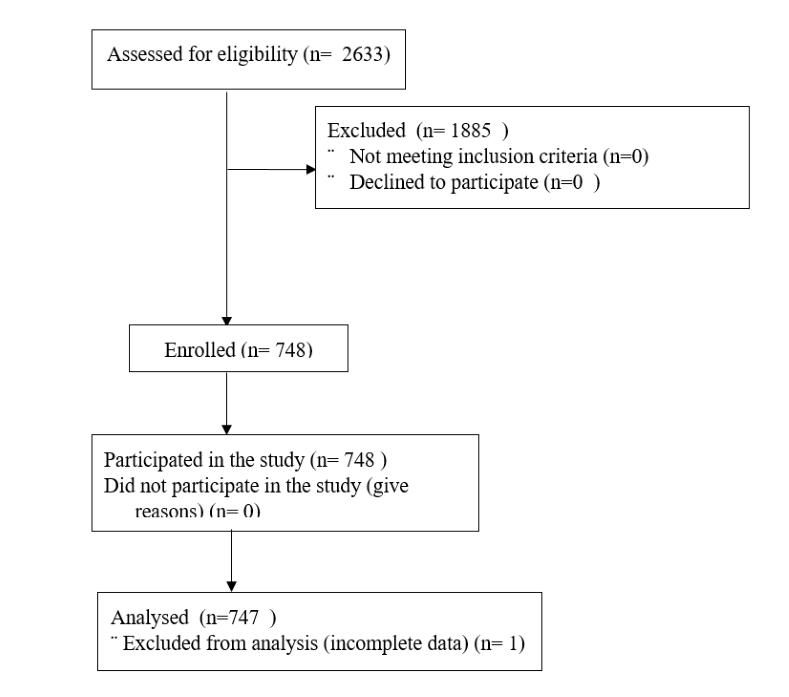

Supplement: S6 File — (TIF) [file pone.0342318.s006.tif]
